# Beak dimensions affect feeding performance within a granivorous songbird species

**DOI:** 10.1242/jeb.249681

**Published:** 2025-03-19

**Authors:** T. Andries, W. Müller, S. Van Wassenbergh

**Affiliations:** ^1^ Laboratory of Functional Morphology, Department of Biology, University of Antwerp, Universiteitsplein 1, 2610 Antwerpen, Belgium; ^2^ Behavioural Ecology and Ecophysiology Research Group, Department of Biology, University of Antwerp, Universiteitsplein 1, 2610 Antwerpen, Belgium

**Keywords:** Domestic canary, Beak kinematics, Beak morphology, Dehusking, Granivory, *Serinus canaria*

## Abstract

Beaks of granivorous songbirds are adapted to dehusk seeds fast and efficiently. This is reflected in the large variety of beak shapes and sizes among species specialized in different seed types. Generally, larger beaks improve the dehusking of larger seeds by transmitting and withstanding higher bite forces. Meanwhile, smaller beaks are better suited for processing smaller seeds by allowing faster beak movements and better seed handling dexterity. These patterns are presumably the result of a trade-off between force and velocity inherent to lever systems. Because beak shape also varies among individuals of the same species, we investigated whether beak shape relates to variation in feeding performance and beak kinematics in the domestic canary (*Serinus canaria*). We analysed beak morphology of 87 individuals through both traditional size measurements and 3D-landmark analysis to capture metrics such as beak depth, length, width and curvature. We related these metrics of morphology to data on feeding performance and beak kinematics during feeding on smaller canary seeds and larger, tougher hemp seeds. We found that individuals with larger absolute beak depths were faster at dehusking the large seeds. Even though individuals with shallow or long beaks displayed higher beak opening–closing frequencies, this did not result in a significantly shorter processing time of the smaller seeds. Our data are therefore in line with the presence of a force–velocity trade-off within a species, but without a velocity-related drawback of beak-size adaptations for increased bite force on the handling performance of a smaller and easier-to-crack seed.

## Teaser

**Summary:** In canaries, individuals with greater beak depth were faster at dehusking large seeds. Unexpectedly, however, individuals with smaller beaks did not perform significantly better at processing smaller seeds.

## INTRODUCTION

The great diversity in shapes and sizes of bird beaks is a well-known example of a morphological adaptation to a wide variety of functions such as feeding, foraging, singing, preening, nest building and more (e.g. Herrel et al., 2009; Moyer et al., 2002; Navalón et al., 2018; Young et al., 2023). Feeding ecology in particular is widely regarded as an important driver of beak shape evolution in taxa such as waterfowl (Olsen, 2017) and songbirds (Gosler, 1987; Benkman, 1988; Bardwell et al., 2001), especially granivorous songbirds, with Darwin's finches as a prime example (Grant et al., 1976; Boag and Grant, 1981; Gibbs & Grant 1987; Herrel et al., 2005a; 2010). Because granivory is a highly complex feeding strategy, involving the dehusking of seeds prior to consumption (Mielke and Van Wassenbergh, 2022; Nuijens and Zweers, 1997), it can be expected that there are strong selective pressures towards beak morphologies that facilitate fast and efficient processing of seeds.

How beak morphology can improve seed processing from a functional perspective has mostly been studied in the context of a trade-off between the ability to generate high bite forces and the ability to move the beak at high speeds (Corbin et al., 2015; Herrel et al., 2009). Firstly, muscle physiology and morphology play a role: birds adapted to feeding on larger and harder seeds typically have larger jaw-closing muscles, which allows them to generate higher bite forces. Consequently, their beaks are adapted to transmit and withstand these higher forces and prevent fracture (Soons et al., 2010, 2015), which is generally reflected by an increase in beak depth and width (Herrel et al., 2005a,b, 2009). Secondly, properties of the jaw lever system and muscle architecture modified to exert high bite forces, such as increased moment arms or pennation angles of the jaw muscles, generally reduce the maximal speed of the jaws (De Schepper et al., 2008; Van Wassenbergh et al., 2005). As such, birds that feed on smaller seeds tend to have longer, shallower and thinner beaks that, together with the muscles that control their movements, allow them to attain higher beak closing velocities (Corbin et al., 2015; Herrel et al., 2009) and reduced handling times of smaller seeds (Grant et al., 1976). Furthermore, van der Meij and Bout (2008) found that a more downward inclination of the beak facilitates a higher bite force. Although Bowman (1961) emphasized the role of beak curvature, posing that more straightened beaks reduce the risk of fracture, finite element modelling of ground finch beaks (Soons et al., 2010) and mathematical approximations of beak curvature (al-Mosleh et al., 2021) suggest the opposite. This suggests that beak shape is more complex beyond the basic dimensions of length, width and depth, and therefore that relationships with bite force and beak velocity are more complex as well.

More in-depth analyses of how beak shape variation relates to jaw closing velocity that go beyond the force–velocity trade-off are hence necessary. A recent study on loggerhead shrikes demonstrated that although there is a force–velocity trade-off during a single biting act at the individual level, this trade-off is not present when correlating peak bite forces with beak closing velocities among individuals (Sustaita and Laurin, 2024). In other words, individuals that can bite harder do not necessarily close their beak slower, or vice versa. This suggests that morphological adaptations that improve bite force and beak velocity do not have to be mutually exclusive and can instead relate to different parts of the jaw system. Indeed, Corbin et al. (2015) already suggested that adaptations that favour speed relate more to lever systems that are efficient in transmitting muscle shortening to beak rotation, whereas bite force is more influenced by adaptations to accommodate for muscle size. Additionally, the kinematics of beak movement involve more than just beak closing velocity (Mielke and Van Wassenbergh, 2022; Andries et al., 2023a). Although many kinematic variables, such as gape distance, beak opening velocity and accelerations, appear strongly correlated with beak closing velocity (Herrel et al., 2009; Andries et al., 2023a), the frequency of beak opening and closing, for example, does not (Andries et al., 2023a). Therefore, it is possible that different aspects of beak kinematics are affected by different morphological adaptations.

Alhough the substantial differences in beak morphology, feeding performance and beak kinematics among granivorous songbirds have probably been an important reason why beak morphology has been considered to be a species-specific trait (e.g. Herrel et al., 2005a,b; van der Meij and Bout, 2008), there is now convincing evidence that beak morphology is also variable at the species level (e.g. Grant, 1981; Heckeberg et al., 2021). This should not be surprising because the costs and benefits of certain trait values likely vary (in a condition- and phenotype-dependent manner) among individuals within a given population. However, direct relationships between beak shape and beak kinematics at the individual level within a single species have, to our knowledge, not been investigated. This is surprising because kinematic capabilities can also vary substantially among individuals of the same species. For example, in canaries, individuals that display the highest beak closing speeds move their beaks about two to three times faster than the slowest individuals (Andries et al., 2023a), and similar ranges of variation have been observed within several species of Darwin's finches (Herrel et al., 2009) and loggerhead shrikes (Sustaita and Laurin, 2024). Yet, it is not clear what causes this broad range in peak closing velocity, or beak kinematics in general, among individuals.

In this study, we investigated how external beak morphology varies among individuals in a population of the domestic Fife fancy canary (*Serinus canaria*) and tested whether this variation is functionally linked to kinematics of beak movement during feeding on two seed types with differing characteristics (relatively small and soft canary seed, and large and tough hemp seed). In addition, we investigated how beak morphology affects feeding performance in general (i.e. seed handling time and the success rate of seed dehusking), as this is what is ultimately ecologically relevant. It also accounts as an integrative measure for aspects of kinematics that are difficult to quantify. Under the assumption of the force–velocity trade-off, we expected that individuals with longer, thinner and more straightened beaks would display higher beak velocities and/or opening–closing frequencies (we cannot state *a priori* whether maximal velocities or frequencies would be more relevant) than individuals with deeper, broader and more curved beaks. Consequently, we expected individuals with longer, thinner and more straightened beaks to be faster and more successful at husking the soft canary seeds. In contrast, individuals with deeper, broader and more curved beaks were assumed to be able to generate higher bite forces and thus should perform better on the large and tough hemp seeds.

## MATERIALS AND METHODS

### Study species and data collection

**Fig. 1.**
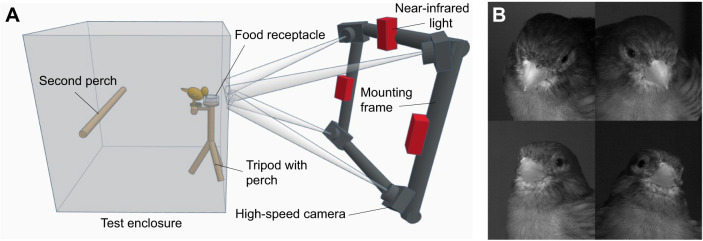
**Experimental setup and example of data output.** (A) Quadroscopic camera setup used to make the recordings as described by Andries et al. (2023a). (B) Example of a set of frames taken from the recordings of Andries et al. (2023a) of a bird with its beak in closed resting position in between feeding bouts. Image contrast was increased to improve visibility.

**Fig. 2.**
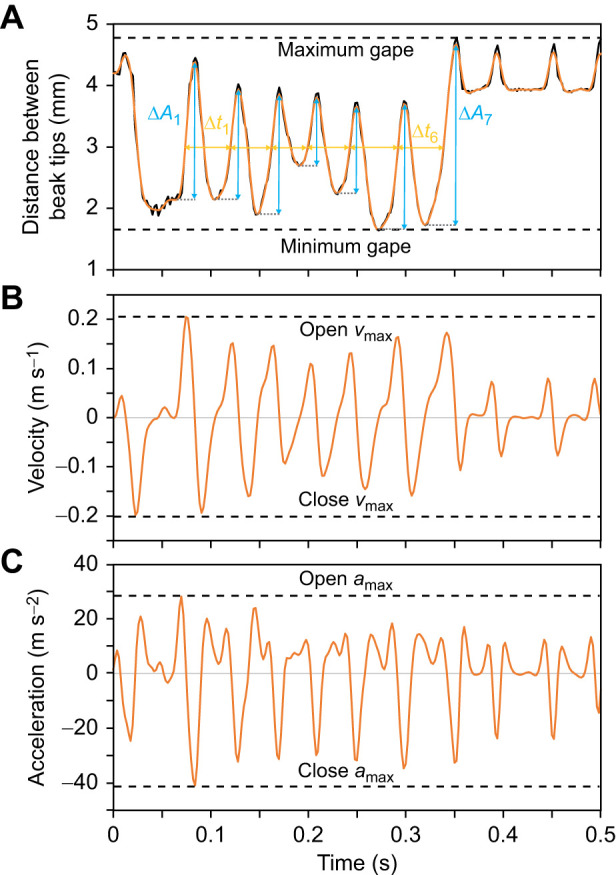
**Illustration of kinematic metrics calculations.** (A) Distance between beak tips of selected fragment after filtering with calculation of maximum gape, minimum gape, average frequency (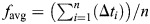) and average amplitude (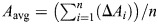). (B) Velocity of the beak tips (calculated as first derivative of the distance) with calculation of maximum opening (open *v*_max_) and closing velocity (close *v*_max_). (C) Acceleration of the beak tips (calculated as second derivative of the distance) with calculation of maximum opening (open *a*_max_) and closing acceleration (close *a*_max_). Figure modified after Andries et al. (2023a).

In a previous study (Andries et al., 2023a) feeding performance, beak kinematic and feeding skill variables were measured in domestic Fife fancy canaries [*Serinus canaria* (Linnaeus 1758)]. Therein, 87 birds (47 males and 40 females) were recorded during feeding on seeds using high-speed cameras in a synchronized quadroscopic setup (Fig. 1A). All individuals in that study belonged to an outbred population housed at the lab and ethical approval for the experiments was granted by the Ethical Committee for Animal Testing of the University of Antwerp (approval number: 2021-35). The synchronized recordings were then used to automatically track the tips of upper and lower beak frame by frame in 3D space, using the machine learning-based tracking software DeepLabCut (Mathis et al., 2018) in conjunction with XMALab (Knörlein et al., 2016), following the workflow of Laurence-Chasen et al. (2020). Fig. 2 demonstrates how kinematic variables were calculated from 3D coordinates of the beak tips, whereas feeding performance metrics were manually measured from the recordings. The resulting data on feeding performance and beak kinematics were directly taken from that study (Andries et al., 2023a,b).

To examine how feeding performance and beak kinematics are linked to beak morphology (present study), we used the video data collected by Andries et al. (2023a) to select single frames of birds with their beak in a closed resting position. This yielded four synchronized images (one per camera) of the head region taken at the exact same point in time per individual (Fig. 1B). These images were calibrated per individual in XMAlab (Knörlein et al., 2016), using footage of a calibration object, consisting of 40 dots at known 3D positions on a 90 deg corner (Andries et al., 2023a). Residual error (a measure of how well the coordinates of the dots of the calibration object on the images line up with the true dimensions of the object) of calibrated images was always less than 2 pixels, or 0.2 mm. This calibration method allowed us to automatically calculate 3D coordinates from points annotated on the previously selected images. This calibration method was successfully applied in previous studies (Andries et al., 2023a; Mielke and Van Wassenbergh, 2022).

### Beak measurements and landmark analysis

**Fig. 3.**
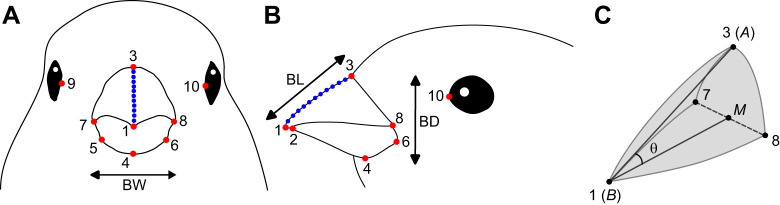
**Schematic representation of landmark placement and beak angle calculation.** Overview of landmark positions and beak measurements in frontal view (A) and lateral view (B). Regular landmarks are indicated in red, semi-landmarks are indicated in blue. (C) Schematic representation of the upper beak to visualize calculation of the upper beak angle (θ). Numbers refer to the same landmarks as depicted in A and B. Letters refer to Eqn 1. Landmarks: (1) tip of the upper beak, (2) tip of the lower beak, (3) central base of the upper beak (base of the culmen), (4) central base of the lower beak (base of the gonys), (5) right base of the lower beak, (6) left base of the lower beak, (7) right corner of the gape, (8) left corner of the gape, (9) frontal end of the right eye, (10) frontal end of the left eye. BD, beak depth; BL, beak length; BW, beak width. Bird head drawings were modified after Mielke and Van Wassenbergh (2022).

To measure beak dimensions, the selected images were first used to annotate 3D landmarks on the external beak (Fig. 3) in XMAlab (Knörlein et al., 2016). Ten regular landmarks were placed as displayed in Fig. 3. Based on the coordinates of these landmarks, we measured beak length, width and depth, as well as the average angle of upper and lower beak (Fig. 3). Beak length was measured as the distance between landmarks 1 and 3, beak width as the distance between landmarks 7 and 8, and beak depth as the distance between landmarks 3 and 4. The angles of the upper and lower beaks were calculated using the properties of the dot product:
(1)

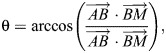
where θ is the beak angle at either the upper or lower beak tip, *A* is the coordinate of landmark 3 for the upper beak and landmark 4 for the lower beak, *B* is the coordinate of landmark 1 for the upper beak and landmark 2 for the lower beak, *M* is the coordinate of the midpoint between landmarks 7 and 8 (for both upper and lower beak), 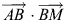 is the dot product of vectors 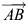 and 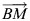, and 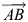 and 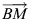 are the lengths of vectors 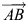 and 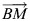, respectively (Fig. 3). We also conducted a geometric morphometrics analysis on the landmark data, but this did not yield much additional biologically relevant insight (see Fig. S1 and Table S1).

To capture beak curvature, 11 semi-landmarks were placed along the culmen (Fig. 3). The curvature of the lower beak was not measured as this was not feasible from our image data. Semi-landmarks were further analysed in R (version 4.3.3) using the package ‘geomorph’ (Baken et al., 2021; https://cran.r-project.org/package=geomorph). First, the semi-landmarks, plus regular landmarks 1 and 3 (Fig. 3) as start and end point of the upper beak's curve, were used to digitize the curves and returned sets of 11 equidistant sliding semi-landmarks (still including landmarks 1 and 3). A generalized Procrustes analysis was conducted to eliminate variation in size, orientation and position as such that only variation in shape remained. Next, a principal component analysis (PCA) was conducted to identify major axes of variation in beak shape. We retained PC axes that explained at least 10% of the total variation (one axis) for further analysis and interpretation.

Data on beak kinematics and feeding performance were taken from Andries et al. (2023a,b). To limit the number of statistical tests, and because many kinematic variables were strongly correlated, we only retained maximum closing velocity and average frequency for our analyses. These variables have been most commonly reported in existing literature (e.g. Corbin et al., 2015; Mielke and Van Wassenbergh, 2022; Sustaita and Laurin, 2024), which facilitates interpretation and discussion of our results. We used total seed handling time and husking success rate as performance variables (Andries et al., 2023a,b).

### Statistical analyses

To test the effects of beak morphology on feeding performance and beak kinematics, we constructed linear mixed models with seed handling time, husking success rate, maximum closing velocity and average frequency as response variables and morphological metrics as predictor variables. Geometric morphometric analysis of the upper beak's curvature yielded one PC axis that explained almost 90% of the variation, which was included as a predictor variable (PC_curve). We included all our beak measurement variables (except one) as predictor variables: beak length, beak depth, beak width and angle of the lower beak. We only excluded angle of the upper beak as including this variable resulted in strong multicollinearity (VIF=10.21), possibly due to moderately high correlations with both beak length (Pearson's *r*=−0.53) and beak depth (Pearson's *r*=0.56). Because we had beak kinematic and feeding performance data of birds feeding on two different seed types (canary seed and hemp), we also included seed type as a predictor and interactions with all other predictor variables. As most birds in our dataset fed on both seed types, we added individual bird identity as random effect. To summarize, we constructed four identical linear mixed models, one per response variable: total seed handling time, husking success rate, maximum closing velocity and average frequency:
(2)

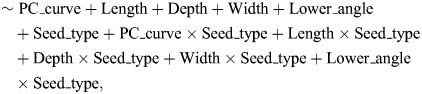
The main predictor variables showed no strong collinearity (highest VIF=1.29). Residuals of all models were checked for normality using QQ plots and heteroscedasticity using residual plots. All statistical analyses were performed in R (version 4.3.3). The packages ‘lme4’ (Bates et al., 2015) and ‘lmerTest’ (Kuznetsova et al., 2017) were used to construct the linear mixed models.

## RESULTS

### Individual variation in beak curvature

**Fig. 4.**
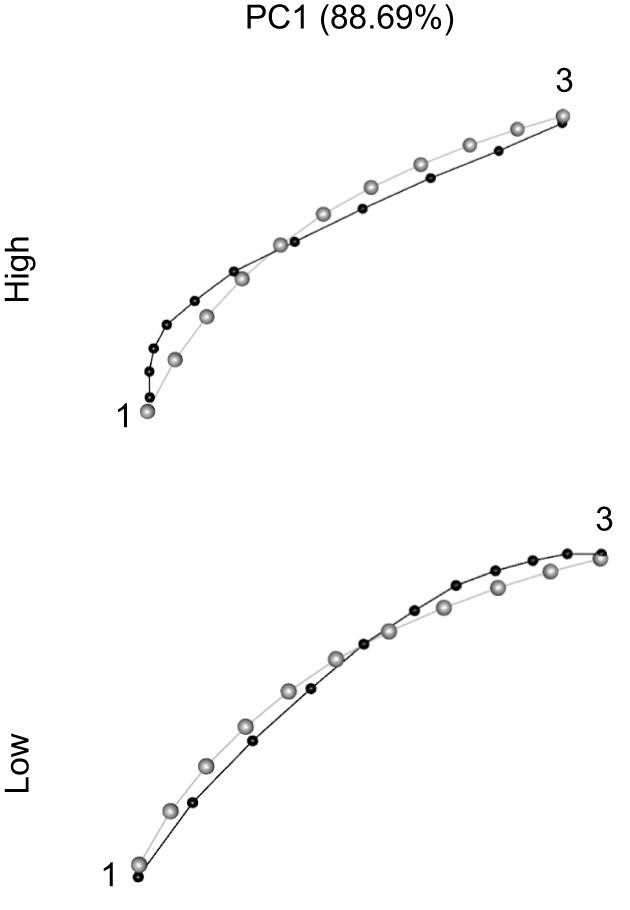
**Wireframe plots representing shape variation captured by the semi-landmark analysis of the upper beak's curvature in lateral view.** Black dots and lines indicate the shape at either high or low values. Grey dots and lines indicate the average shape. Numbers refer to the landmarks as described in the Materials and Methods and Fig. 3.

Variation in the curvature of the upper beak as captured by the PCA analysis is shown in Fig. 4. PC1 explained almost all variation (88.69%). This PC was mainly composed of where along the beak most of the curvature was located. At high values, the beak curved most near the tip (landmark 1), whereas at low values most of the curvature was situated near the base of the beak (landmark 3). Summary statistics of the PCA analysis can be found in Table S1.

### Effects of beak size and shape on feeding performance and beak kinematics

**Fig. 5.**
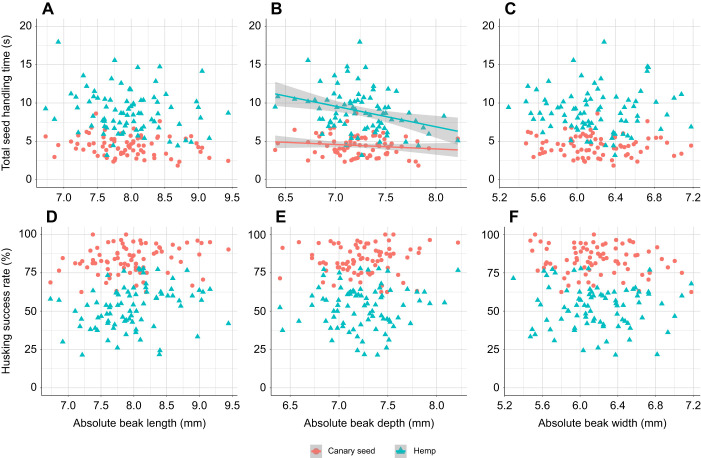
**Linear regressions of performance metrics on absolute beak sizes.** Linear relationships of absolute beak sizes with seed handling time (A–C) and the success rate of seed husking (D–F) during feeding on canary seed (*N*=79) and hemp seed (*N*=82). Data points represent individual birds and are the mean value of 10 feeding trials. Significant relationships are represented by a regression line and its 95% confidence interval shaded in grey. Numerical and statistical results of the regression analyses can be found in Table S2. Relationships of PC1 of the semi-landmark analysis and the lower beak angle with seed handling time and success rate can be found in Fig. S2A–D.

**Fig. 6.**
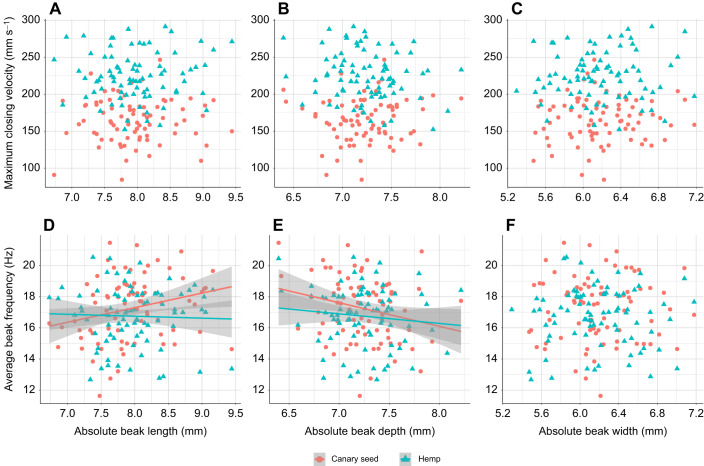
**Linear regressions of kinematic variables on absolute beak sizes.** Linear relationships of absolute beak sizes with maximum beak closing velocity (A–C) and the average frequency of beak opening–closing (D–F) during feeding on canary seed (*N*=79) and hemp seed (*N*=82). Data points represent individual birds and are the mean value of five feeding trials. Significant relationships are represented by a regression line and its 95% confidence interval shaded in grey. Numerical and statistical results of the regression analyses can be found in Table S2. Relationships of PC1 of the semi-landmark analysis and the lower beak angle with maximum closing velocity and average frequency can be found in Fig. S2E–H.

Beak depth had a significant effect on seed handling time in interaction with seed types (depth×seed type, *P*=0.023). Individuals with the deepest beaks processed hemp seeds approximately 5 s faster on average than individuals with the shallowest beaks. Meanwhile, such an effect was practically absent during feeding on canary seeds (Fig. 5B). Beak depth also had an effect on average frequency in interaction with seed types (depth×seed type, *P*=0.018). The average frequency of beak opening and closing decreased with 2–3 Hz between individuals with the shallowest compared with those with the deepest beaks during feeding on canary seed, whereas feeding on hemp seeds only saw a decrease of 1–2 Hz (Fig. 6E). Average frequency was significantly affected by beak length in interaction with seed types (length×seed type, *P*=0.001). Average frequency was 2–3 Hz greater in individuals with the longest beaks when compared with individuals with the shortest beaks during feeding on canary seed, but this difference was close to zero when feeding on hemp seed (Fig. 6D). Beak width (Figs 5 and 6), angle of the lower beak (Fig. S2) and PC1 of the upper beak's curvature (Fig. S2) were not significantly correlated with any performance or kinematic variables. The success rate of seed husking (Fig. 5D,F) and maximum closing velocity of the beak (Fig. 6A–C) were not significantly correlated with any measure of morphology (see also Table S2).

## DISCUSSION

The relationship between beak morphology and feeding performance in songbirds is well known at the species level (e.g. Boag and Grant, 1981; Gosler, 1987; Herrel et al., 2010), but our study is the first to investigate how variation in beak shape and size among individuals of the same species can affect the kinematics of beak movement. We captured external beak morphology in a population of canaries through traditional beak size measurements, combined with a landmark analysis of the beak's curvature. We found noteworthy effects of absolute beak dimensions (traditional size measurements) on the frequency of beak opening–closing, as well as seed handling time. In contrast, we found no evidence that maximum closing velocity was affected by any measure of morphology.

### Beak dimensions affect feeding performance

Beaks of granivorous songbirds are regarded to be well adapted to process seeds fast and efficiently. In other words, birds with deeper beaks can generate higher bite forces and are expected to be better at cracking larger seeds (Herrel et al., 2005a,b, 2009), whereas birds with longer beaks can attain higher velocities and should thus be more efficient at husking smaller seeds (Corbin et al., 2015; Herrel et al., 2010). Our results confirm these predictions, though not to the degree we expected. Birds with deeper beaks were substantially faster at husking hemp seeds, but we saw no noteworthy effects of beak size on the handling of canary seeds (Fig. 5B).

Under the assumption that deeper beaks are associated with larger jaw closing muscles (Herrel et al., 2005b), it is expected that birds with deeper beaks are better at husking hemp seeds. Hemp seeds are relatively large, require considerable force to be cracked open (van der Meij et al., 2004; van der Meij and Bout, 2006) and hence appear rather difficult to dehusk for canaries (Andries et al., 2023a; Kear, 1962). Being able to exert higher bite forces should reduce the number of cracking attempts needed to successfully crack the seed husk, which could be beneficial because most of the time handling hemp seeds is spent on positioning the seed between upper and lower beak alternated by cracking attempts (Andries et al., 2023a; Mielke and Van Wassenbergh, 2022). Indeed, having a deeper beak reduces, as we show, the total time needed to process a seed (see also van der Meij et al., 2004).

Furthermore, we expected that birds with longer, thinner beaks would be faster or more successful at husking canary seeds, but found no clear evidence for such patterns. A possible explanation might be related to the characteristics of canary seeds. On the one hand, canary seeds are notably thinner than hemp seeds (mean±s.d. width: canary seed: 1.84±0.23 mm; hemp seed: 2.85±0.33 mm), but they are actually similar in length to hemp seed (mean±s.d. length: canary seed: 4.67±0.40 mm; hemp seed: 4.23±0.40 mm), thus overall still relatively large (Andries et al., 2023a). Yet the advantages of thinner and longer beaks that can attain higher velocities only come into play when feeding on smaller seeds (Corbin et al., 2015; Herrel et al., 2009). On the other hand, canary seeds are open-shelled seeds, which require considerably less force to dehusk than close-shelled seeds such as hemp seed (van der Meij et al., 2004; van der Meij and Bout, 2006). Hence, it is possible that the advantages of deeper and longer beaks cancel out during feeding on canary seeds. This suggests that the effects of beak morphology on feeding performance are contingent on the interplay between seed size and strength (and possibly more seed characteristics).

### Beak dimensions affect average frequency, but not peak closing velocity

Surprisingly, we found no evidence that beak shape or size affected maximum closing velocity in canaries. It is possible that the morphology of the external beak alone is not sufficiently linked with aspects that affect beak closing speed. It has traditionally been hypothesized that how well the beak's lever system is adapted to moving fast is determined by the ratio of in- and out-lever length (Corbin et al., 2015; Herrel et al., 2009), also called the mechanical advantage of the lever system (Kammerer et al., 2005; Westneat, 2003). However, bird skulls are highly kinetic (e.g. the upper beak also moves upward during seed handling, see Mielke and Van Wassenbergh, 2022), so considering them as a simple one-lever system (i.e. mandible rotating about the quadrate joint) is an oversimplification. But perhaps even more relevant than the mechanical advantage might be the geometric and physiological properties of the jaw adductor muscles (Labonte, 2023). As the jaw adductors' force and work capacity for biting presumably makes them overdeveloped for the purpose of powering beak closure, beak speed is expected to be limited mainly by the gearing properties of the musculoskeletal system (Polet and Labonte, 2024). Therefore, characteristics that are expected to be the prime determinants of beak closing speed are the moment arms of the jaw adductors, the pennation angles of the jaw adductors and the maximum contraction speeds of the muscle fibres. Either way, neither the mechanical advantage of the beak's lever system nor the orientation and physiological properties of the jaw adductors can be derived from external beak morphology alone, and thus could unfortunately not be measured in our sample.

**Fig. 7.**
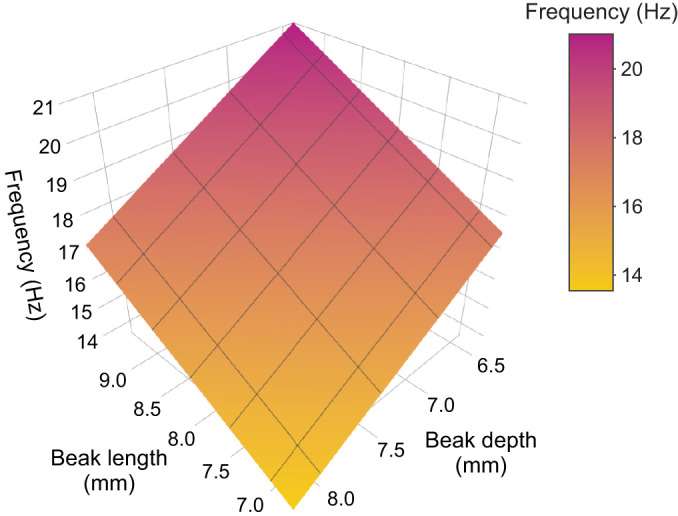
**Bilinear least-squares regression of absolute beak length and depth against average beak opening–closing frequency during feeding on canary seeds (*R*²=0.1508) for illustration purpose.** The colour gradient represents the values of frequency for a given beak length and depth. Black lines represent the grid lines for the beak length and beak depth axes.

In contrast, however, beak size affected the average frequency of beak opening and closing during seed handling, though only during feeding on canary seed. Although the observed effects were not particularly strong, it is striking how beak length and depth have opposite effects on frequency, where frequency increased with increasing beak length, but decreased with increasing beak depth (Figs 6D,E, 7). These effects of beak length and depth are expected in the case of a force–velocity trade-off (Corbin et al., 2015; Herrel et al., 2005a,b, 2009), as birds with longer beaks are opening and closing their beaks faster (and vice versa for birds with deeper beaks). However, this assumption requires elaboration, because differences in frequency could also arise as a result of differences in amplitude. That is, a bird that moves its beak at the same speed but opens its beak wider (because it might be feeding on larger seeds, for example) will have a lower opening–closing frequency. Yet, this seems unlikely, as Andries et al. (2023a) reported that average frequency and amplitude are not strongly correlated in canaries. Hence, individual variation in frequency of beak opening and closing in canaries is most likely related to variation in average beak opening and closing speed.

This apparent contradiction where beak length and depth affect average frequency (and thus average beak speed), but not maximum closing velocity, was unexpected. However, Andries et al. (2023a) observed a trade-off between maximal beak closing velocity and seed husking success rate, i.e. a speed–accuracy trade-off (Fitts, 1954; Heitz, 2014). This suggests that it might not be optimal to just maximize speed, as this leads to higher chances of making mistakes and dropping seeds instead, which is similar to the finding that many animals rarely move at maximal speeds (Nasir et al., 2017; Wynn et al., 2015). It is therefore likely that the maximal closing velocity displayed by individual birds during feeding is not necessarily a matter of only morphological or physiological constraints, but also includes a plastic, behavioural component. Meanwhile, the frequency of beak opening–closing displayed during feeding, being an averaged measure over time, might more closely approach the maximal beak speeds that individuals are physically capable of achieving, and maintaining, during the act of feeding. However, the impact of behavioural variability is likely still substantial.

### A note on individual variation in beak curvature

The idea that the curvature of bird beaks can have an impact on the performance of feeding on seeds dates back to Bowman (1961). More recent studies on Darwin's finches found that more curved beaks are generally better suited for feeding on larger and harder seeds and that it reduced the risk of fracture (Herrel et al., 2010; Soons et al., 2010; al-Mosleh et al., 2021). In this study, we analysed the curvature of the upper beak to investigate whether it could also be related to feeding performance and beak kinematics in canaries. Curiously, most variation in beak curvature among individuals in our study was not found in the strength of the curvature, but rather in whether most of the curving occurred near the tip or the base of the beak (Fig. 4). To our knowledge, this type of variation in curvature has not been demonstrated before in granivorous songbirds. Yet, at least in the context of our study, it may be an epiphenomenon, as it does not seem to affect seed feeding performance or the speed of beak movements.

It is important to note that, because we investigated external beak shape, the observed curvatures are of the rhamphotheca, the keratinous sheath that envelops the bony beak itself. Although the rhamphotheca plays a role in stress dissipation during biting (Soons et al., 2012, 2015), its curvature does not necessarily line up with the curvature of the bony beak underneath (Urano et al., 2018). Soons et al. (2015) also found that the rhamphotheca was thicker in areas of the beak that are expected to experience greater stresses during biting in some species of Darwin's finches. This, coupled with the fact that the rhamphotheca is a plastic and continuously growing structure, might suggest that the variation we observed in beak curvature could be caused by variation in rhamphotheca thickness formed in response to differing stresses during biting. For example, it could be possible that the more pronounced curvature near the tip of the beak in some individuals is formed by a locally thicker rhamphotheca, because these individuals hold seeds closer to the tip of the beak during seed cracking, resulting in greater forces being exerted more often in that area. Future research investigating whether individuals do consistently differ in the positioning of seeds relative to the beak during cracking could shed more light on whether individual variation in beak curvature does have a functional benefit.

As a final remark, it might be worth mentioning that domestication might have had an effect on beak shape, such as on the shape of the rhamphotheca via beak overgrowth (Speer and Powers, 2016) or metabolic bone diseases (Ranjan et al., 2018). Even though we did not notice this in our sample, this could potentially affect the shape or strength of the beak and, consequently, feeding performance.

### Conclusions

Our study shows that variation in beak morphology can affect the performance and kinematics of seed feeding among individuals of the same species. Individuals with deeper beaks were faster at dehusking hemp seeds, and a force–velocity trade-off appeared to be present during feeding on canary seeds, where individuals with deeper beaks opened and closed their beaks at lower frequencies, whereas individuals with longer beaks displayed higher beak frequencies. Hence, despite the presence of a force–velocity trade-off, beaks that are adapted for increased bite force (i.e. deeper beaks) do not appear to incur a decrease in performance during feeding on easier-to-crack seeds.

Interestingly, maximum beak closing speeds do not seem to be affected by beak size metrics or shape. This suggests that external beak shape alone is insufficiently linked with factors affecting peak beak movement speeds during feeding. Alternatively, some individuals may on average close their beak at submaximal speeds to retain a sufficiently high precision in repositioning the seed. Further research should investigate individual variation in internal beak morphology, particularly the orientation and physiological properties of the jaw-closing muscles and test how these affect the beak opening and closing speeds that birds can achieve, in order to test our hypotheses.

## Supplementary Material

10.1242/jexbio.249681_sup1 Supplementary information
