# Supplementary material for: Beak dimensions affect feeding performance within a granivorous songbird species
Source: J Exp Biol. 2025 Mar 19;228(6):jeb249681. doi: 10.1242/jeb.249681 (PMC11959705; doi:10.1242/jeb.249681)
Supplement: Supplementary information [file jexbio-228-249681-s1.pdf]

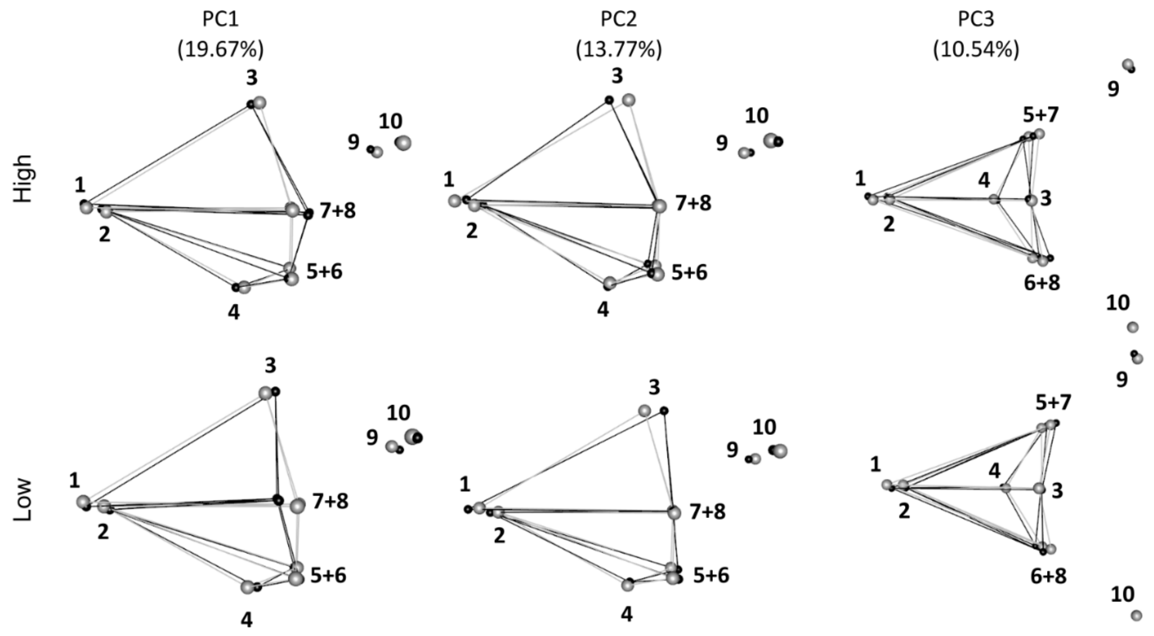

Fig. S1. Wireframe plots representing shape variation captured by the first three PCs of the regular landmark analysis (PC1, PC2 and PC3). Plots of PC1 and PC2 are shown in lateral view, plots of PC3 in dorsal view. Percentages between parentheses indicate the proportion of total variation explained by the respective PC. Black dots and lines indicate the shape at either high (first row) or low (second row) values of the respective PCs. Grey dots and lines indicate the average shape. Numbers refer to the landmarks as described in the MATERIALS & METHODS and Fig. 3.

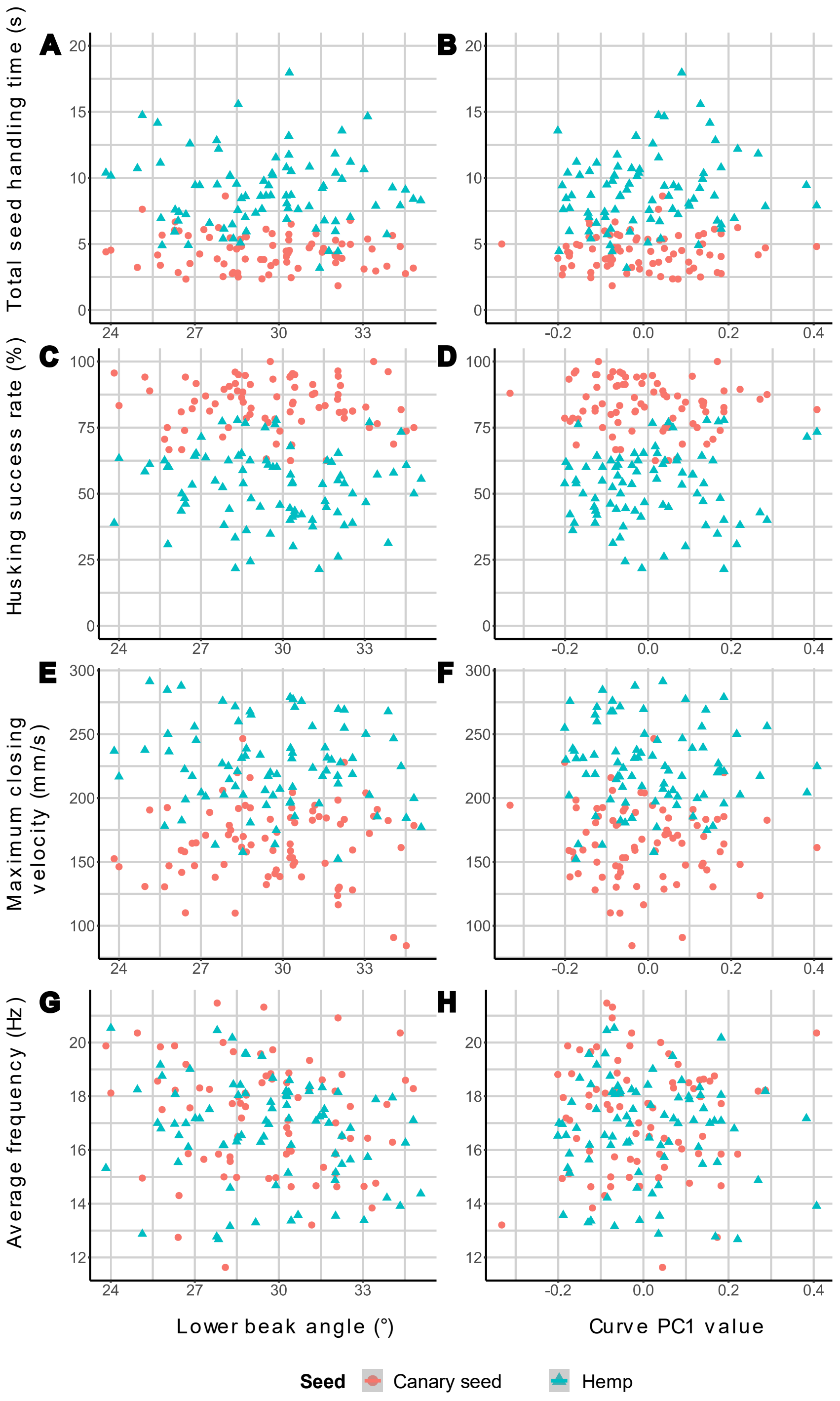

Fig. S2. Linear relationships of lower beak angle and PC1 of the semi-landmark analysis of the curvature of the upper beak with seed handling time (A-B), husking success rate (C-D), maximum beak closing velocity (E-F) and the average frequency of beak opening-closing (G-H) during feeding on canary seed (N = 79) and hemp seed (N = 82). Data points represent individual birds and are the mean value of either 10 feeding trials (total seed handling and success rate) or 5 feeding trials (maximum closing velocity and frequency). Numerical and statistical results of the regression analyses can be found in Table S2.

Table S1. Summary statistics of the PCAs of the regular landmark analysis to capture overall beak shape and the semi-landmark analysis to capture the curvature of the upper beak. The first 10 PC-axes are reported.

	Overall beak shape (Fig. S1)		Curve upper beak (Fig. 4)	
**PC axis**	**Eigenvalues**	**Proportion of variance**	**Eigenvalues**	**Proportion of variance**
**1**	0.00105	0.1967	0.01909	0.8869
**2**	0.00073	0.1377	0.00125	0.0580
**3**	0.00056	0.1054	0.00087	0.0405
**4**	0.00043	0.0803	0.00011	$5.0156*10^{-3}$
**5**	0.00040	0.0754	$7.5030*10^{-5}$	$3.4860*10^{-3}$
**6**	0.00038	0.0715	$3.0416*10^{-5}$	$1.4131*10^{-3}$
**7**	0.00034	0.0634	$2.1427*10^{-5}$	$9.9551*10^{-4}$
**8**	0.00026	0.0498	$1.6380*10^{-5}$	$7.6103*10^{-4}$
**9**	0.00018	0.0334	$1.2029*10^{-5}$	$5.8900*10^{-4}$
**10**	0.00016	0.0297	$9.4971*10^{-6}$	$4.4124*10^{-4}$

Journal of Experimental Biology • Supplementary information

Table S2. Summary statistics of the independent variables per regression model. Significant p-values are indicated in bold.

Variable	Slope estimate	Standard error	t-value	p-value
**Handling time (s)**				
PC1_curve	0.326	1.825	0.179	0.858
Length (mm)	-0.664	0.484	-1.371	0.172
Depth (mm)	-0.150	0.770	-0.195	0.845
Width (mm)	-0.133	0.633	-0.211	0.834
Lower_angle (°)	-0.124	0.101	-1.230	0.221
Seed_type	12.975	9.914	1.309	0.195
PC1_curve* Seed_type	3.904	2.534	1.540	0.128
Length*Seed_type	0.071	0.674	0.105	0.917
Depth*Seed_type	-2.464	1.061	-2.321	**0.023***
Width*Seed_type	1.078	0.879	1.227	0.224
Lower_angle* Seed_type	0.071	0.140	0.510	0.612
**Success rate (%)**				
PC1_curve	-12.042	10.182	-1.183	0.239
Length (mm)	4.390	2.701	1.625	0.106
Depth (mm)	1.107	4.296	0.258	0.797
Width (mm)	-5.760	3.531	-1.631	0.105
Lower_angle (°)	0.050	0.564	0.089	0.929
Seed_type	-69.746	55.955	-1.246	0.215
PC1_curve* Seed_type	18.629	14.298	1.303	0.195
Length*Seed_type	2.764	3.809	0.726	0.469
Depth*Seed_type	-1.423	5.991	-0.238	0.813
Width*Seed_type	4.196	4.962	0.845	0.399
Lower_angle* Seed_type	0.033	0.789	0.041	0.967

**Maximal closing velocity (mm/s)**				
PC1_curve	-13.702	27.872	-0.492	0.624
Length (mm)	-1.527	7.396	-0.206	0.837
Depth (mm)	8.871	11.761	0.754	0.452
Width (mm)	3.796	9.667	0.393	0.695
Lower_angle (°)	0.049	1.544	0.032	0.974
Seed_type	227.639	147.345	1.545	0.126
PC1_curve* Seed_type	7.032	37.707	0.186	0.853
Length*Seed_type	5.238	10.008	0.523	0.602
Depth*Seed_type	-28.007	15.776	-1.775	0.080
Width*Seed_type	2.420	13.041	0.186	0.853
Lower_angle* Seed_type	-0.722	2.073	-0.348	0.729
**Frequency (Hz)**				
PC1_curve	0.576	1.657	0.348	0.729
Length (mm)	1.342	0.443	3.026	**0.003***
Depth (mm)	-2.172	0.701	-3.099	**0.002***
Width (mm)	0.172	0.578	0.297	0.767
Lower_angle (°)	0.031	0.092	0.335	0.738
Seed_type	5.324	6.948	0.766	0.446
PC1_curve* Seed_type	-0.806	1.792	-0.450	0.654
Length*Seed_type	-1.579	0.467	-3.378	**0.001***
Depth*Seed_type	1.796	0.744	2.413	**0.018***
Width*Seed_type	-0.108	0.609	-0.177	0.860
Lower_angle* Seed_type	-0.191	0.097	-1.963	0.054

Journal of Experimental Biology • Supplementary information
